# Comparison of the efficacy of aescin and diclofenac sodium in the management of postoperative sequelae and their effect on salivary Prostaglandin E2 and serum C–reactive protein levels after surgical removal of impacted mandibular third molar: a randomized, double-blind, controlled clinical trial.

**DOI:** 10.12688/f1000research.145643.3

**Published:** 2024-10-30

**Authors:** Anuroop Singhai, Rajanikanth Kambala, Nitin Bhola

**Affiliations:** 1 Oral Surgery, General Dentistry Program, Batterjee Medical College, Jeddah, Makkah, 21442, Saudi Arabia; 2 Oral and Maxillofacial Surgery, Datta Meghe Institute of Higher Education and Research, Wardha, Maharashtra, 442001, India

**Keywords:** Aescin, Diclofenac, pain, swelling, C-reactive protein, prostaglandin E2, third molar

## Abstract

**Introduction:**

Surgical removal of an impacted third molar is one of the most common oral surgical procedures performed in dental offices. The postoperative phase is often associated with severe inflammation. Non-steroidal anti-inflammatory drugs (NSAIDs) are usually prescribed to manage postoperative discomfort. NSAIDs have been associated with gastrointestinal bleeding, renal function disturbances, and platelet count reductions. Thus, the present study demonstrates the utility of aescin in managing postoperative discomfort after the surgical removal of impacted mandibular third molars.

This study aimed to correlate and compare the impact of aescin and diclofenac on salivary PGE2 levels and serum C-reactive protein levels after surgical extraction of the mandibular third molar. The study will also evaluate and compare the effectiveness of individual drug therapy in managing postoperative pain, swelling and mouth opening.

**Methods:**

The planned study is a single-center, double-blind, randomized, parallel, prospective clinical trial. Each patient will be prescribed either diclofenac sodium 150 mg/day or aescin (escin) 120 mg/day to be taken orally in divided doses for five days after surgically removing the impacted mandibular third molar.

Pain will be assessed using a visual analog scale. Facial swelling and mouth opening will be recorded using a metric scale with standardized reference points. ELISA (enzyme-linked immunosorbent assay (ELISA) will be employed to measure salivary Prostaglandin E2 and serum C–reactive protein levels. All parameters will be recorded preoperatively (T0) on the second postoperative day (T1) and fifth postoperative day (T2).

**Conclusion:**

The proposed study is expected to show a clinically acceptable response to the administration of aescin for the management of postoperative discomfort compared to diclofenac sodium after third molar surgery.

The proposed study is expected to positively manipulate the levels of salivary Prostaglandin E2 and serum C–reactive protein, which are reliable inflammatory markers.

The outcome of this study may provide an efficacious and safe alternative to conventional nonsteroidal anti-inflammatory drugs for managing postoperative discomfort following third molar surgery.

## Introduction

### Background and rationale

The backbone of most oral surgical practices worldwide is the extraction of impacted third molars. It is the most common oral surgical procedure ^1^ performed in dental offices. However, the postoperative phase is often concomitant with severe pain, reduced mouth opening, and marked facial inflammation. Most patients are prescribed corticosteroids and nonsteroidal anti-inflammatory drugs (NSAIDs) to manage their postoperative discomfort. NSAIDS have been associated with gastrointestinal bleeding, disturbance in renal function, and a reduction in platelet count. ^2^ Certain newer NSAIDs like lornoxicam have been evaluated for their efficacy in managing pain after third molar surgery due to lesser incidences of gastrointestinal lesions as compared to other NSAIDs. ^3^ However, this untoward still poses a challenge to clinicians.

Because of these limitations, many phytotherapeutic alternatives have been suggested. Aescin (Escin) has generated interest among researchers over the years. It is a practical part of Aesculus hippocastanum and was first isolated in 1953. It combines triterpene saponins and has demonstrated anti-inflammatory, antiedematous, and venotonic properties in various preparations. ^4^ Thus, the present study demonstrates the utility of aescin in managing postoperative discomfort after the surgical removal of impacted mandibular third molars.

Another concern over the years has been the selection of NSAIDs, which predominantly depends on subjective factors, including the surgeon’s preference and personal experience. The need for an hour is to use a measurable marker of inflammation that can be quantitatively controlled by drug administration. Prostaglandin E2 (PGE2) is a reliable marker of inflammation, which has been isolated from various body fluids, including saliva. ^5^ Owing to inflammation, C-reactive protein (CRP) emerges in the blood. Various studies have used CRP concentrations to monitor the healing process after drug therapy because of its sensitivity. ^6^ This study aimed to correlate salivary PGE2 levels and serum C-reactive protein levels with subjective and objective symptoms after surgical extraction of the mandibular third molar and their relationship with therapeutic use of aescin and diclofenac. The objectives of the study is to compare the efficacy of aescin and diclofenac for managing pain, swelling, mouth opening and the need for rescue analgesics. The study will also evaluate the patient satisfaction regarding each drug therapy.

### Background and rationale: choice of comparators

Diclofenac was chosen for comparison because it has dose equivalence to aescin and is widely used to manage postoperative sequelae associated with third molar surgery.

## Protocol

### Aim of the study

To compare the efficacy of aescin (escin) and diclofenac in managing postoperative sequelae following surgical removal of impacted mandibular third molars and their effects on salivary Prostaglandin E2 and serum C-reactive protein levels.

## Objectives

The objectives of the present study are as follows:

1. To compare the efficacy of aescin and diclofenac sodium in managing postoperative sequelae following surgical removal of impacted mandibular third molars.
2. To compare the efficacy of aescin and diclofenac sodium in manipulating salivary Prostaglandin E2 (PGE2) and serum C-reactive protein (CRP) levels in patients undergoing surgical removal of the impacted mandibular third molar.
3. To assess patient satisfaction regarding therapy.
4. To evaluate the need for rescue analgesics in the postoperative period.

### Trial design

A single-center, double-blind, randomized, parallel, prospective clinical trial will be conducted at the Sharad Pawar Dental College, Datta Meghe Institute of Higher Education and Research (DMIHER), Sawangi, Wardha.

Two groups will be created:

Group A – Diclofenac sodium

Group B – Aescin (escin)

The randomization process will be performed using a computer-generated subject allocation list.

Trial participants and outcome assessors will be blinded. The study will be a double-blinded clinical trial where the participant and the principal investigator will not be aware of the drug being used in the postoperative period. The double-blinded study minimizes the risk of various types of biases, such as observer bias or confirmation bias, which may influence the results of the investigation.

### Study setting

A total of 50 patients presenting to the outpatient department of oral and maxillofacial surgery, Sharad Pawar Dental College and Hospital, Sawangi, Wardha, India, with impacted mandibular third molars with a definitive indication for extraction will be enrolled in the study.

### Eligibility criteria

*Inclusion criteria*

1. Patients giving informed written consent.
2. Age group 18-40 yrs. The age range is selected to have homogeneity, reduced comorbidities, relevance to the target population and better baseline functionality.
3. ASA I and healthy ASA II physical status.
4. Patients presenting with impacted mandibular third molars that are indicated for removal.
5. Slightly or moderately difficult impacted mandibular third molars, according to the Pederson difficulty index with the difficulty score in the range of 3 to 7.

*Exclusion criteria*

1. Presence of oral mucosal comorbid conditions that may affect salivary inflammatory markers.
2. Patients not reporting for follow-up as per study protocol.
3. Presence of systemic infections that may alter the levels of serum C-reactive protein.
4. Presence of acute infection that necessitates the use of antibiotic therapy.
5. Known allergies to drugs under study and local anesthetic agents.
6. Patients with a history of xerostomia or hypersalivation.

## Methods

### Interventions: description

A clinical trial will be conducted with approval from the institutional ethics committee. The Consolidated Standards of Reporting Trials (CONSORT) statement guidelines will be used for this study.

Patients will be prescribed either diclofenac sodium 150 mg/day or aescin (escin) 120 mg/day ^7^ to be taken orally in divided doses for five days. Each patient will be subjected to one of the drugs after the procedure.

Saliva collection will be done using a sterile polystyrene tube. A kit specific to PGE2 will be used to measure PGE2 levels in each specimen. ^5^

Venous blood will be subjected to a C-reactive protein (CRP) test to measure C-reactive protein ^8^ levels in blood serum.

Saliva and serum samples will be processed in Central Research Laboratory.

### Surgical procedure

All patients will be administered 1 g of oral amoxicillin 1 h before the procedure. The standard surgical protocol for the removal of impacted mandibular third molars under local anesthesia will be followed.

### Interventions: modifications

Drug therapy will be discontinued in cases of adverse reactions reported by the patient.

### Interventions: adherence

Patients will be recalled on the second and fifth postoperative days. The number of tablets remaining will be calculated to assess compliance with the trial protocol.

### Interventions: concomitant care

Patients will be required to adhere to the standard postoperative instructions following extraction.

Tramadol 50 mg will be prescribed as a rescue analgesic for the postoperative period.

## Outcomes

Postoperative sequelae after surgical removal of the impacted mandibular third molar will be estimated by assessing pain, facial swelling, and mouth opening.

Pain: The visual analog scale (VAS) ^9^ will be used to assess pain on a scale ranging from 0 to 10. The worst imaginable pain will be graded as 10, and the absence of pain will be graded as 0.

Facial swelling will be measured using a metric tape and standard reference points. ^7^ The following reference points will be used for the measurement:

• Soft tissue pogonion - Tragus
• The angle of the mandible - Corner of the eye
• Corner of mouth - Tragus

Mouth opening: The maximum interincisal distance will be recorded using a metric scale with standardized ruler and a digital Vernier caliper. ^10^

Salivary Prostaglandin E2 and serum C-reactive protein levels will be measured by ELISA (enzyme-linked immunosorbent assay (ELISA) preoperatively (T0) on the second postoperative day (T1) and fifth postoperative day (T2).

Patient satisfaction regarding therapy: Study subjects will classify their experience from 1 to 5, where 1 = poor, 2 = fair, 3 = good, 4 = very good, and 5 = excellent.

The need for rescue analgesics will be evaluated by counting the number of tramadol capsules or tablets required by the patient until the fifth postoperative day (T2).

### Participant timeline

Pain, facial swelling, mouth opening, serum C-reactive protein, and salivary prostaglandin E2 levels will be recorded preoperatively (T0) on the second postoperative day (T1) and fifth postoperative day (T2).

Patient satisfaction regarding therapy and the need for rescue analgesics will be recorded on the fifth postoperative day.

### Sample size

The study will be a non-inferiority trial with a continuous outcome.

The sample size was established based on data obtained from a previous study conducted by Salgia et al. ^11^ Serum C-reactive protein level was chosen as the primary outcome variable.

Formula using mean difference

$$n1=n2=2\frac{{\left(Z_{\alpha }+Z_{\beta }\right)}^{2}\sigma^{2}}{{\left(\delta \right)}^{2}}$$

$$Z_{\alpha }=1.96\;$$

α = Type I error at 5% at both sides two tailed

Z _β_ = 0.84 = Power at 80%

Primary Variable = CRP (C-reactive protein) levels

The mean difference after treatment CRP (mean ± SD) for the diclofenac group was 16.305 ± 3.977 (as per the reference article). ^10^

Considering 20% clinically relevant margin for aescin = ( 16.305∗20)/100=3.26

Sample size:

$$N=n1=n2=2\frac{{\left(1.96+0.84\right)}^{2}{\left(3.977\right)}^{2}}{{\left(3.261\right)}^{2}}=23$$

Assuming 10% dropout = 2

Total sample size required = 23 + 2 = 25 each group.

Thus, a total sample size of 50 subjects presenting with impacted mandibular third molars will be enrolled in the study.

### Recruitment

All patients presenting to the outpatient unit of the Oral and Maxillofacial Department and fulfilling the enrolment criteria of the study will be recruited. The principal investigator coordinated with each patient to achieve the desired sample size.

## Methods: Assignment of interventions (for controlled trials)

### Allocation: sequence generation

Study participants will be randomized using the SCIENCER application. As a specific age range was chosen for the study, further stratification and subset creation will not be performed.

### Allocation concealment mechanism

All the patients will be sequentially numbered for the purpose of allocation concealment.

### Allocation: implementation

The principal investigator will then enrol the participants. The research supervisor allocate the sequence and assign the participants to the intervention.

### Blinding

Trial participants and the principal investigator will be blinded.

### Blinding (masking): emergency unblinding

Unblinding will be performed if a patient reports adverse side effects to the emergency unit during the follow-up period.

### Data collection plan

Demographic, clinical, and laboratory data for each patient will be collected using the JOTFORM application.

### Data collection plan: retention

Telephonic reminders will be administered to each patient, according to the follow-up protocol. The research supervisor will directly dispense medication to the participants to promote retention.

### Data management

Demographic, clinical, and laboratory data will be segregated in the JOTFORM application based on patient codes. The filtered data will be downloaded and stored in an Excel spreadsheet for statistical analysis.

### Statistical analysis plan

***Statistics: outcomes***

***Statistics: additional analyses***

***Statistics: analysis population and missing data***

*Primary variable*

Inferential statistics will be used to compare the two groups for measurement scores, resulting in their mean change in a primary variable (visual analog scale, facial swelling, mouth opening, salivary prostaglandin E2, serum C-reactive protein) between baseline and end visits.

Baseline variables will be tested for significance in the mean using ANOVA or Kruskal-Wallis test for more than two assessment periods. A post-hoc (Tukey’s or Duncan’s) test will be used to find a significant difference between the two groups for pair-wise comparison.

*Outcome variables*

Outcome variables will be tested for intra-difference in measurement at pre- and post-visits using a paired t-test to determine the mean’s significance. The unpaired t-test for comparison of two groups and ANOVA for comparison of three groups will be used for intergroup differences.

Statistical analysis will be carried out using R-software free version.

## Methods: monitoring

### Data monitoring: formal committee

Data monitoring will be carried out under the aegis of DSCC at DMIHER, Sawangi, and Wardha.

### Data monitoring: interim analysis

The interim analysis and review will be carried out by a member of the institutional ethics committee. Trial termination rights were reserved by the Institutional Ethical Committee.

### Harms

Telephonic conversations will be conducted prior to follow-up visits to inquire about any untoward events. In case of any adverse events, patients will be managed at the institutional hospital, and the necessary care will be rendered free of cost.

All adverse events will be reported to the Institutional Ethical Committee.

### Auditing

An interim audit of the trial will be conducted by the research supervisor. A final audit will be carried out by nominated members of the DSCC at the DMIHER, Sawangi, and Wardha.

### Ethics and dissemination

*Research ethics approval*

Ethical approval has been obtained from the Institutional Review Board of the Datta Meghe Institute of Medical Sciences (now renamed as Datta Meghe Institute of Higher Education and Research) Sawangi, Wardha. (Reference number: DMIMS (DU)/IEC/2022/842 on 05/04/2022).

### Protocol amendments

Amendments to the protocol will be carried out only after approval from the Institutional Ethical Committee and DSCC at DMIHER, Sawangi, Wardha.

### Consent or assent

The Principal investigator will be required to answer any queries by the patient regarding the trial, and will be responsible for obtaining informed consent.

### Consent or assent: ancillary studies

No biological specimen will be stored for ancillary studies and hence, ancillary study consent is not applicable.

### Confidentiality

Access to information from individual participants will be restricted to the principal investigator and research supervisor.

### Declaration of interests

There are no financial or competing interests for the principal investigators for the overall trial or each study site.

### Data access

The principal investigator and research supervisor will be custodian of the final trial data. The IEC and DSCC have access to the final trial dataset.

### Ancillary and post-trial care

The trial will be conducted in accordance with good clinical practice and a strong adverse event-monitoring protocol. The safety of the procedures and drugs used in this study has been established. However, in case of any harm, compensation will be carried out according to institutional policy.

### Dissemination policy: trial results

This clinical trial will be published as an original research article in an indexed journal. Open access release of the trial results will be performed to ensure optimum exposure of the scientific community.

### Dissemination policy: authorship

The final trial data will be published as postdoctoral research (PHD thesis). The authorship associated with this trial will be limited to the principal investigator and research supervisor, with acknowledgements to individuals involved at various stages of the trial.

### Dissemination policy: reproducible research

The PHD thesis will be made public after approval from DSCC.

### Study status

The study is currently in the stage of a pilot project.

## Discussion

There is growing interest in medical fraternity to find safer alternatives to NSAIDs. Despite advances in pharmaceutical technology, the pharmacokinetic properties of NSAIDs, such as diclofenac, have improved, however the dose-dependent gastrointestinal severe, renal, and cardiovascular adverse effects remain a cause of concern with the use of NSAIDs.

Previous studies have indicated that phytotherapeutic drugs have a favorable impact on inflammatory parameters and are safe for managing pain and discomfort following surgical removal of the third molar. Aescin (escin) is a phytotherapeutic drug that has been recognized for its anti-inflammatory properties. Previous studies have also suggested the glucocorticoid-like activity of aescin along with its gastroprotective action. However, further research is needed to examine the effectiveness of aescin in managing postoperative pain, facial swelling, and mouth opening following surgical removal of the third molar.

Isola et al. (2024) conducted a parallel-arm, randomised clinical trial on forty-two periodontitis patients to compare the outcome of minimally invasive non-surgical therapy (MINST) with quadrant-wise subgingival instrumentation (Q-SI) on C-reactive protein (CRP) and lipoprotein-associated phospholipase A2 (Lp-PLA2) levels, and their association with clinical implications. The study suggested that lower levels of C-reactive protein and Lp-PLA2 were seen in patients receiving MINST after 1-year follow-up. ^12^

Marciano et al. (2024) present a compelling narrative review that elucidates the remarkable clinical benefits of aescin in modulating the bradykinin pathway. They draw attention to a critical gap in scientific literature, noting a scarcity of human studies on aescin’s effects. Alarmingly, no comparative research has been conducted between aescin—a natural compound—and the mentioned nutraceuticals. Overall, aescin stands out as a drug with an impressive safety profile and versatile pharmacodynamics, positioning it for optimal anti-inflammatory and anti-edema effects through bradykinin pathway suppression. However, to fully unlock its potential, robust human studies are essential to assess aescin’s impact on bradykinin. ^13^

Singh et al. (2021) conducted a study on 90 patients that aimed to compare the efficacy of diclofenac sodium and mefenamic acid in managing pain after mandibular impacted third molar surgery and to assess changes in C-reactive protein (CRP) levels as an inflammation marker. The study involved surgical removal using standardized procedures, with patients divided into two groups: one receiving diclofenac sodium (50 mg) and the other mefenamic acid (500 mg) for three days. Pain was evaluated using the visual analog scale (VAS), revealing a mean VAS of 2.58 for diclofenac and 3.46 for mefenamic acid (p < 0.05). Postoperative CRP levels increased significantly in both groups. However, diclofenac showed lower CRP levels compared to mefenamic acid. The findings suggested that diclofenac sodium is effective for pain relief and that CRP evaluation can serve as a useful indicator of postoperative inflammation. ^14^

Nowak et al. (2021) conducted a study of 60 patients to assess the impact of A-PRF application during third molar surgery and its effect on C-reactive protein concentrations and wound healing. Patients who received A-PRF after the procedure had a faster rate of decreased CRP levels. They also suggested that A-PRF application significantly reduced the symptoms of regional inflammation and incidence of dry socket. ^6^

In a detailed review of escin, Gallelli (2019) suggested that it has potent anti-inflammatory, antiedematous, venotonic, and endothelial protective properties. They described the basic mechanism of action of escin. Oral escin and its topical gel have been demonstrated to increase venous tone and reduce edema, resulting in quantifiable clinical progress in patients with blunt injury and chronic venous insufficiency. ^4^

Isola et al. (2018) conducted a clinical trial of eighty-two patients needing mandibular third molar extraction surgery. The study participants were randomly allocated into three groups. Group 1, placebo; Group 2, ibuprofen; Group 3, a phytotherapeutic drug (baicalin 190 mg, bromelain 50 mg, and escin 30 mg). All medications were prescribed twice daily for five days after tooth extraction. A visual analog scale was used to assess pain. A metric scale was used to record alterations in facial contours and mouth opening in millimeters. The outcome of this clinical trial implies that phytotherapeutic drug therapy effectively manages postoperative pain and discomfort. ^9^

Graziani et al. (2017) enrolled 40 patients in a case-control study demonstrating systemic inflammation following third molar removal. Patients who underwent third molar removal had more significant systemic inflammation, oxidative stress, and triglyceride levels than controls, and the first postoperative week had increased WBC counts and serum C-reactive protein and fibrinogen levels. The baseline values of the markers were observed three months after extraction. Higher systemic inflammation is associated with impacted third molars, and their elimination may signify a novel method for investigating acute inflammation and defining favorable systemic outcomes in humans. ^8^

Mehra et al. (2013) conducted a double-blind, randomized clinical trial of 80 patients to assess the effect of four different pharmacological regimens on prostaglandin E2 (PGE2) levels in urine and saliva. Saliva and urine samples were collected at planned intervals. After removing the impacted lower third molars, the findings were compared with clinical progression. Patients who received ibuprofen fared significantly better in terms of most parameters. They further concluded that PGE2 is measurable in saliva and urine, and its rise and fall can be detected in both saliva and urine. They further suggested that PGE2 correlates with subjective and objective clinical symptomatology and that administration of anti-inflammatory drugs manipulates its levels in the body. ^5^

The current scientific literature must be more extensive in demonstrating the correlation of the drug’s efficacy with reliable inflammation markers and associated clinical symptomatology.

Considering this, the present study will be a randomized, double-blind, non-inferiority trial to compare the efficacy of aescin and diclofenac sodium in the management of postoperative pain, swelling, and mouth opening, as well as their effects on salivary Prostaglandin E2 and serum C-reactive protein levels after surgical removal of the impacted mandibular third molar. The trial will be conducted as per SPIRIT guidelines. ^15^

In conclusion, the proposed study is expected to positively manipulate the levels of salivary Prostaglandin E2 and serum C–reactive protein, which are reliable inflammatory markers. The study may show clinically acceptable responses in the management of postoperative pain, swelling and mouth opening with administration of aescin. The outcome of this study may provide an efficacious and safe alternative to conventional nonsteroidal anti-inflammatory drugs for managing postoperative discomfort following third molar surgery.

## Trial registration

www.ctri.nic.in

Trial Registration Number is: CTRI/2023/09/058004

## Trial registration: data set

Not applicable

## Protocol version

v1 dated 26/07/2023

Identifier: Dr. Rajanikanth Kambala

## Roles and responsibilities: sponsor and funder

Study planning, data collection, research management, analysis, preparation of report and publication

## Roles and responsibilities: committees

The study will be carried out under the aegis of the Institutional Ethical Committee and the DSCC at DMIHER, Sawangi, Wardha, Maharashtra, India.

## Data Availability

No data is associated with this article. **Zenodo:** Extended data sheet for “Comparison of the efficacy of aescin and diclofenac sodium in the management of postoperative sequelae and their effect on salivary Prostaglandin E2 and serum C–reactive protein levels after surgical removal of impacted mandibular third molars: a randomized, double-blind, controlled clinical trial.”. Zenodo. https://doi.org/10.5281/zenodo.10605612. ^15^ This project contains the following extended data:
• Model Consent form • Biological specimen • Patient Case Record form Zenodo: SPIRIT checklist for “Comparison of the efficacy of aescin and diclofenac sodium in the management of postoperative sequelae and their effect on salivary Prostaglandin E2 and serum C–reactive protein levels after surgical removal of impacted mandibular third molars: a randomized, double-blind, controlled clinical trial.” https://doi.org/10.5281/zenodo.10605565. ^16^ Data are available under the terms of the Creative Commons Attribution 4.0 International license (CC-BY 4.0).
