# Supplementary material for: Comparison of the efficacy of aescin and diclofenac sodium in the management of postoperative sequelae and their effect on salivary Prostaglandin E2 and serum C–reactive protein levels after surgical removal of impacted mandibular third molar: a randomized, double-blind, controlled clinical trial
Source: F1000Res. 2024 Oct 30;13:106. Originally published 2024 Feb 16. [Version 3] doi: 10.12688/f1000research.145643.3 (PMC11538593; doi:10.12688/f1000research.145643.3)
Supplement: Peer review history

# Reviewer report: Reviewer response for version 3

**DOI:** 10.5256/f1000research.173977.r336513

**Published:** 2024-11-06

**Authors:** Gaetano Isola

**Affiliations:** 1 University of Catania, Catania, Italy

The authors have well modified the protocol based on the issues raised by the reviewer. The protocol can be indexed in this form.

Is the study design appropriate for the research question?

Yes

Is the rationale for, and objectives of, the study clearly described?

Partly

Are sufficient details of the methods provided to allow replication by others?

Partly

Are the datasets clearly presented in a useable and accessible format?

Yes

Reviewer Expertise:

Oral surgery, periodontitis

I confirm that I have read this submission and believe that I have an appropriate level of expertise to confirm that it is of an acceptable scientific standard.

# Reviewer report: Reviewer response for version 3

**DOI:** 10.5256/f1000research.173977.r336514

**Published:** 2024-11-05

**Authors:** Ahmed Saaduddin

**Affiliations:** 1 Mansoura University, Mansoura, Egypt

I appreciate the efforts of the authors in addressing the feedback from the initial review. Your revisions have significantly strengthened the overall clarity and impact of the paper.

 I was particularly impressed by the following improvements:

1. **Expanded discussion**: The inclusion of recent studies enhances the context of your work and demonstrates a thorough engagement with existing literature. This addition enriches your arguments and positions your research within the current discourse.
2. **Methodological rigour**: The clarifications regarding your methodology address earlier concerns and add credibility to your objectives. It is now evident how your approach effectively supports your conclusions.

Overall, I am satisfied with the revisions made and believe the article is now ready for indexing. I commend your responsiveness to feedback and your commitment to enhancing the quality of your work.

Is the study design appropriate for the research question?

Yes

Is the rationale for, and objectives of, the study clearly described?

Yes

Are sufficient details of the methods provided to allow replication by others?

Partly

Are the datasets clearly presented in a useable and accessible format?

Not applicable

Reviewer Expertise:

I am an Oral and Maxillofacial Surgeon whose primary research is in the field of minor oral surgery, clinical implantology and dental traumatology.

I confirm that I have read this submission and believe that I have an appropriate level of expertise to confirm that it is of an acceptable scientific standard.

# Reviewer report: Reviewer response for version 2

**DOI:** 10.5256/f1000research.167297.r332167

**Published:** 2024-10-24

**Authors:** Ahmed Saaduddin

**Affiliations:** 1 Mansoura University, Mansoura, Egypt

**Objectives**

 The authors have clearly defined objectives with a focus on both subjective (pain, satisfaction) and objective (PGE2, CRP levels) outcomes. Including additional patient-reported outcomes, such as satisfaction with therapy.

**Study Design**

 The protocol outlines a single-center, double-blind, randomized, parallel, prospective clinical trial, which is appropriate for this type of comparative study. The use of a non-inferiority design is justified, especially since the aim is to assess aescin as a safer alternative to diclofenac.

**Methods**

- **Participants:** The inclusion and exclusion criteria are comprehensive. However, the rationale for the age restriction (18-40 years) should be elaborated. Why not include a broader age range?
- **Randomization and Blinding:** The randomization process using a computer-generated allocation is a robust method. However, further details on the blinding process (e.g., how blinding will be maintained) would add clarity.

**Discussion and Literature Review**

 The literature review provides good context for the study, though the author can add suggested references that are more current and directly related to the specific outcomes being measured.

**Overall, Clarity and Structure**

 The protocol is well-structured and generally clear.

 However, it could benefit from adding the appropriate responses to the queries raised in the above sections.

Is the study design appropriate for the research question?

Yes

Is the rationale for, and objectives of, the study clearly described?

Yes

Are sufficient details of the methods provided to allow replication by others?

Partly

Are the datasets clearly presented in a useable and accessible format?

Not applicable

Reviewer Expertise:

I am an Oral and Maxillofacial Surgeon whose primary research is in the field of minor oral surgery, clinical implantology and dental traumatology.

I confirm that I have read this submission and believe that I have an appropriate level of expertise to confirm that it is of an acceptable scientific standard, however I have significant reservations, as outlined above.

## References

[1] : Assessment of C-reactive Protein Level and Efficacy of Diclofenac Sodium and Mefenamic Acid in Relieving Pain in Mandibular Impacted Third Molar Surgery. *J Contemp Dent Pract* .2021;22(1) :39-41 34002707

[2] : Escin's Action on Bradykinin Pathway: Advantageous Clinical Properties for an Unknown Mechanism?. *Antioxidants (Basel)* .2024;13(9) : 10.3390/antiox13091130 10.3390/antiox13091130 39334789 PMC11429163

# Response

**Published:** 2024-10-24

**Authors:** Anuroop Singhai

**Affiliations:** Oral and Maxillofacial Surgery, Sharad Pawar Dental College, Datta Meghe Institute of Higher Education and Research, Wardha, Maharashtra, 442001, India, Wardha, Maharashtra, India

Dear Dr. Ahmed

 Thank you for your insightful comments and constructive feedback on our manuscript. We appreciate the time you took to evaluate our study, and we have addressed your points as follows:

**Participants:** Thank you for highlighting the need for clarification regarding our age restrictions. We have elaborated on this rationale in the revised manuscript, explaining that our focus on the 18-40 age range is intended to minimize variability related to age-related physiological differences and comorbidities that could affect the study outcomes.

**Randomization and Blinding:** We acknowledge that additional details on our blinding process would enhance clarity. We have included a more thorough explanation of how blinding will be maintained throughout the study to ensure the integrity of the results.

**Discussion and Literature Review:**

 We appreciate your feedback on the literature review. We have incorporated the studies suggested by you in the discussion section in our study to provide a stronger context for our findings.

 Thank you for your positive remarks regarding the clarity and structure of our protocol. We have tried to address all queries appropriately in the revised manuscript.

 We believe that these revisions will strengthen our manuscript and better address the valuable concerns you have raised. Thank you once again for your thorough review and support in improving our work. We look forward to submitting our revised manuscript for your consideration.

# Reviewer report: Reviewer response for version 2

**DOI:** 10.5256/f1000research.167297.r288083

**Published:** 2024-06-08

**Authors:** Gaetano Isola

**Affiliations:** 1 University of Catania, Catania, Italy

In this revised version of the protocol, the authors have well addressed all issues raised by the reviewer. The protocol can be indexed.

Is the study design appropriate for the research question?

Yes

Is the rationale for, and objectives of, the study clearly described?

Partly

Are sufficient details of the methods provided to allow replication by others?

Partly

Are the datasets clearly presented in a useable and accessible format?

Yes

Reviewer Expertise:

Oral surgery, periodontitis

I confirm that I have read this submission and believe that I have an appropriate level of expertise to confirm that it is of an acceptable scientific standard.

# Reviewer report: Reviewer response for version 1

**DOI:** 10.5256/f1000research.159617.r272570

**Published:** 2024-05-22

**Authors:** Gaetano Isola

**Affiliations:** 1 University of Catania, Catania, Italy

In the manuscript entitled: " Comparison of the efficacy of aescin and diclofenac sodium in the management of postoperative sequelae and their effect on salivary Prostaglandin E2 and serum C–reactive protein levels after surgical removal of impacted mandibular third molar: a randomized, double-blind, controlled clinical trial" the authors aimed to assess salivary PGE2 levels and serum Creactive protein levels with subjective and objective symptoms after surgical extraction of the mandibular third molar and their relationship with drug therapy.

 The proposed study is expected to show a favorable response to the administration of aescin for the management of postoperative discomfort compared to diclofenac sodium after third molar surgery.

 The proposed study is expected to positively manipulate the levels of salivary Prostaglandin E2 and serum C–reactive protein, which are reliable inflammatory markers.

 Major comments:

 In general, the idea and innovation of this study regards the analysis of perioperative effects of third molar and biomarkers is interesting and novel because the role these aspects in medicine are validated but further studies on this topic could be an innovative issue in this field could be open a creative matter of debate in literature by adding new information.

 The study was well conducted by the authors; However, there are some concerns to revise that are described below.

 The introduction section resumes the existing knowledge regarding the important factor linked with the impact mediators involved together with biomarkers in dentistry.

 However, as the importance of the topic, the reviewer recommends to update the literature through read, discuss some recent interesting articles, that helps the authors to better introduce and discuss the role of CRP in periodontal treatment (similar to third molar) and on NSAIDs approaches (Isola G, et al., 2024 [Ref 1]) , (Isola G, et al., 2019 [Ref 2])

 The authors should be better specified, at the end of the introduction section, the rationale of the study and the aim of the protocol. In the central section, should better clarify inclusions and exclusions criteria of the selected sample.

 Please better state the results obtained in the abstract.

 The discussion section appears well organized with the relevant paper that support the conclusions, even if the authors should better discuss the relationship regarding the periodontitis and risk of oxidative stress evolution and periodontal treatment that could improve the quality of life in oral diseases patients with impacted third molar. The conclusion should reinforce in light of the discussions.

 In conclusion, I am sure that the authors are fine clinicians who achieve very nice results with their adopted protocol.

 Minor Comments:

 Abstract:

- Better formulate the abstract section by better describing the aim of the study

Introduction:

- Please refer to major comments

Discussion

- Please add a specific sentence that clarifies the results obtained in the first part of the discussion

Is the study design appropriate for the research question?

Yes

Is the rationale for, and objectives of, the study clearly described?

Partly

Are sufficient details of the methods provided to allow replication by others?

Partly

Are the datasets clearly presented in a useable and accessible format?

Yes

Reviewer Expertise:

Oral surgery, periodontitis

I confirm that I have read this submission and believe that I have an appropriate level of expertise to confirm that it is of an acceptable scientific standard, however I have significant reservations, as outlined above.

## References

[1] : Effects of minimally invasive non-surgical therapy on C-reactive protein, lipoprotein-associated phospholipase A2, and clinical outcomes in periodontitis patients: A 1-year randomized, controlled clinical trial. *J Periodontol* .2024; 10.1002/JPER.23-0518 10.1002/JPER.23-0518 38696664

[2] : Analysis of the Effectiveness of Lornoxicam and Flurbiprofen on Management of Pain and Sequelae Following Third Molar Surgery: A Randomized, Controlled, Clinical Trial. *J Clin Med* .2019;8(3) : 10.3390/jcm8030325 10.3390/jcm8030325 30866586 PMC6463050

# Response

**Published:** 2024-05-30

**Authors:** Anuroop Singhai

**Affiliations:** Oral and Maxillofacial Surgery, Sharad Pawar Dental College, Datta Meghe Institute of Higher Education and Research, Wardha, Maharashtra, 442001, India, Wardha, Maharashtra, India

Dear Dr. Isola

 Thank you for taking the time to review our manuscript submitted to F1000research. We greatly appreciate your valuable feedback and constructive comments, which have undoubtedly enhanced the quality of our work.

 We have carefully considered each of your suggestions and have made the necessary revisions to address the concerns raised. Below, we outline the changes made in response to your comments:

1. Introduction Section:
  - We have provided a more comprehensive rationale for the study and explicitly stated the aim of the protocol at the end of the introduction section. This aims to offer readers a clear understanding of the objectives and purpose of our research.
2. Methods Section:
  - In the central section, we have meticulously clarified the inclusion and exclusion criteria of the selected sample. This ensures transparency and assists readers in understanding the criteria used for participant selection.
3. Abstract:
  - We have revised the abstract however as this is a study protocol in preliminary stage and data collection is still going on. So the results will be published after the completion of study.
4. Discussion Section:
  - We have reorganized the discussion section to further elucidate the relationship between periodontitis, periodontal therapy and inflammatory markers. Additionally, we have incorporated relevant literature to support our conclusions effectively.
5. Conclusion:
  - The conclusion has been strengthened to reinforce key insights gleaned from the discussion. By aligning the conclusion with the broader implications discussed, we aim to provide readers with a more robust and conclusive summary of our study approach and relevance.

We are grateful for your acknowledgment of our clinical expertise and the positive assessment of our adopted protocol. Your encouraging words serve as motivation to continually strive for excellence in our research endeavors.

 Once again, we extend our sincere gratitude for your insightful review and invaluable input. We believe that the revisions made have significantly improved the manuscript and hope that it now meets the standards for publication in F1000research.

 Thank you for your continued support and consideration.
